# Thoracoscopy-assisted modified Nuss procedure for the treatment of pectus excavatum in children: a retrospective single-center experience

**DOI:** 10.3389/fped.2026.1793429

**Published:** 2026-07-02

**Authors:** Xiaolong Chen, Li Shen, Hongbin Zhu, Wenjun Qin, Yingying Xiao, Hao Shi, Aling Xie, Haifa Hong

**Affiliations:** Department of Cardiothoracic Surgery, Shanghai Children’s Hospital, School of Medicine, Shanghai Jiao Tong University, Shanghai, China

**Keywords:** children, experience, modified Nuss procedure, pectus excavatum, thoracoscopy

## Abstract

**Objectives:**

This research aimed to evaluate the safety and efficacy of the thoracoscopy-assisted modified Nuss procedure for the treatment of pectus excavatum in children at our hospital.

**Methods:**

A retrospective analysis was conducted on patients with pectus excavatum who underwent surgery between January 2023 and June 2025 at our hospital. In all, 199 patients underwent the thoracoscopy-assisted modified Nuss procedure (MP group), whereas 32 underwent the thoracoscopy-assisted traditional Nuss procedure (TP group). Additionally, the patients were divided into two groups based on the number of bars implanted: the single-bar group (SB group) and the double-bar group (DB group).

**Results:**

A total of 231 patients were included, comprising 176 males and 55 females. The age range was 3.00–18.00 years, with a median of 12.00 years (interquartile range [IQR]: 10.00–14.00 years). The postoperative hospital stay ranged from 3.00 to 18.00 days, with a median of 6.00 days (IQR: 5.00–8.00 days). The incidence of surgical complications was low (8.65%). Each case showed an improvement in Haller index (HI), and both the children and their parents were satisfied with the postoperative chest appearance. Statistical analysis showed that significant differences were found between the MP and TP groups in operation time [median 65.00 min (IQR 55.00–65.00 min) vs. 87.50 min (80.00–93.75 min), *P* < 0.001], postoperative pain score [6.00 (5.00–6.00) vs. 6.00 (5.00–7.00), *P* < 0.05], and postoperative hospital stay [6.00 days (5.00–7.00 days) vs. 9.00 days (7.25–11.00 days), *P* < 0.001]. In addition, significant differences were observed between the SB and DB groups in age [12.00 years (10.00–13.00 years) vs. 4.10 years (3.56–4.90 years), *P* < 0.001], operation time [65.00 min (55.00–65.00 min) vs. 95.00 min (90.00–101.25 min), *P* < 0.001] and postoperative hospitalization time [6.00 days (5.00–7.00 days) vs. 7.50 days (6.00–11.00 days), *P* < 0.05].

**Conclusions:**

Our results showed that compared to the traditional Nuss procedure, the thoracoscopy-assisted modified Nuss procedure achieved a comparable correction of the HI while offering shorter operation time, lower postoperative pain score, and shorter postoperative hospital stay, which may be suitable for pediatric patients with pectus excavatum.

## 1 Introduction

Pectus excavatum is the most common congenital chest wall deformity, with an estimated incidence of 1 in 700–1,000 live births (1). This deformity is not uncommon worldwide, particularly in China. Although the malformation can be symmetric, it is more frequently asymmetric and may involve other parts of the thorax. Pectus excavatum can be identified at birth or may become apparent later in childhood. Its etiology remains unclear; possible causes include overgrowth of the costal cartilage resulting in posterior sternal displacement (2). Furthermore, pectus excavatum has a genetic predisposition (3). In some cases, it may be associated with other conditions such as connective tissue disorders and congenital heart disease (4). A minority of secondary pectus excavatum cases may occur following thoracic surgery, especially in children, potentially related to the serratus anterior muscle (5). The consequences of this defect encompass reduced exercise tolerance, growth restriction due to cardiac compression, restrictive lung disease, and cosmetic deformities accompanied by psychological distress, including depression and diminished self-esteem (6, 7).

For mild cases of pectus excavatum, conservative treatment may be adopted, with reported good outcomes using the Vacuum Bell device (8). However, the majority of pectus excavatum cases are managed surgically. In 1998, Nuss introduced a minimally invasive procedure for the correction of pectus excavatum, which demonstrated favorable results (9). To date, the Nuss procedure has proven to be a convenient and effective treatment; however, the insertion or removal of the bar can sometimes be difficult and traumatic, and certain complications remain (10–12). First, since the bars used in the conventional Nuss procedure lack a pre-formed curved segment, they must be shaped intraoperatively using special tools, which prolongs operative time. Second, the process of flipping the bar is often challenging and invasive. Finally, fixation relying solely on wires may provide insufficient stability, while both placement and removal of the bar are time-consuming, posing considerable challenges to the surgeon (13, 14).

**Figure 1:**
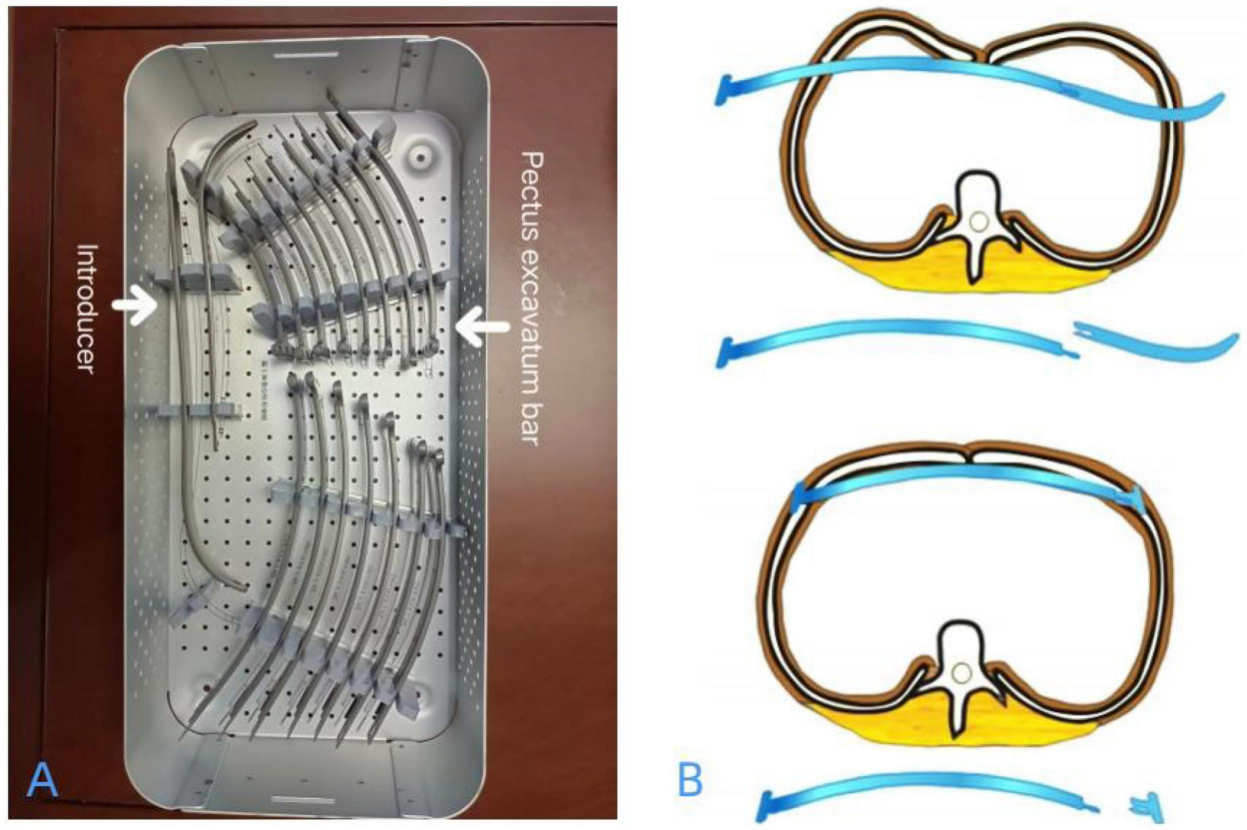
The material object of the pectus excavatum bar and introducer **(A)** the demonstration diagram of the modified nuss procedure **(B)**.

In this article, we introduce a modified Nuss procedure, which we call the thoracoscopy-assisted modified Nuss procedure. This novel modified technique was initially proposed by Dr. Li Guoqing in 2015 (15), and has been further refined recently at our institution. We applied a modified Nuss bar (Shanghai Puwei Medical Instrument, Shanghai, China) for treating pectus excavatum. Compared with the previous steel bar reported in 2015, we adjusted the material and the specifications. The type of bar is made of titanium alloy, which exhibits excellent biocompatibility and high mechanical strength. The bars are curved according to the normal human anterior chest wall and have many large and small sizes according to different lengths. The preset curvature of each titanium bar model was derived from a large-scale anthropometric database of healthy anterior chest wall morphology. For every length increment, the specific curvature profile was calculated to match the statistical mean curvature of a normal chest wall for the corresponding thoracic size, ensuring an anatomically optimized fit for the general population. The small sizes are often used for children, and the large sizes are often used for adolescents. The bars have 18 different lengths, varying from 13 to 30 cm. Each specification has a 1 cm difference. One end of the bar is fused with a stabilizer, and the other end is connected with the introducer or stabilizer. The introducer and stabilizer are of different sizes to match the bars of different sizes. Specifically, bars measuring 13–20 cm in length were paired with a standard-sized introducer and stabilizer, whereas bars measuring 21–30 cm utilized a proportionally larger, yet structurally identical, model. The introducer is used to guide the bar through the chest. Once the bar passes through the chest and is located, the introducer is replaced with a stabilizer, which transfers the supporting force from the intercostal muscles to the ribs (Figure 1).

**Figure 2:**
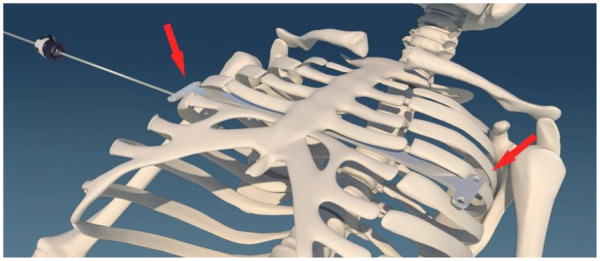
The demonstration diagram of the pectus excavatum bar and chest, indicated by the red arrow, shows the bilateral rib supporting points.

Compared to the thoracoscopy-assisted traditional Nuss procedure, the key advantages of this modified approach are as follows: First, the newly designed bar is made of titanium alloy and is pre-formed and pre-attached to the introducer prior to surgery, eliminating the need for intraoperative bending. This avoids potential damage to the bar and reduces the risk of pectus recurrence. Second, the introducer-bar complex can be inserted or removed using a push-or-pull mechanism without extensive flipping, thereby simplifying the procedure and reducing intraoperative trauma. Third, unlike the traditional method in which the bar is secured only with wires, the new design incorporates screw holes that allow for more stable fixation using screws and locking clips. Finally, in this procedure, the bar is primarily supported by the ribs rather than the intercostal muscles, which effectively alleviates postoperative pain and reduces complications such as bar displacement caused by intercostal muscle tearing (Figure 2). There are few published studies reporting the use of the modified Nuss procedure in pediatric patients. This article aims to evaluate the safety and efficacy of this procedure at our hospital.

## 2 Materials and methods

### 2.1 Patients

Between January 2024 and June 2025, a total of 231 children diagnosed with pectus excavatum were treated at the Department of Cardiothoracic Surgery, Shanghai Children's Hospital and were included in this retrospective cohort study (Table 1). The cohort consisted of 176 males and 55 females, with ages ranging from 3.00 to 18.00 years. Age was non-normally distributed (Shapiro–Wilk *p* < 0.001) and presented as median (IQR). The median age of the cohort was 12 years (IQR 10–14 years). We conducted a retrospective analysis of clinical data from 231 patients with pectus excavatum, focusing on surgical procedures, treatment outcomes, and prognosis. Prior to surgery, all patients underwent chest computed tomography (CT) for measurement of the Haller index, electrocardiography, and color Doppler echocardiography to evaluate cardiac function, as well as anteroposterior and lateral spinal radiographs to assess the presence of scoliosis. In all cases, 199 patients underwent the thoracoscopy-assisted modified Nuss procedure and 32 patients underwent the traditional Nuss procedure.

**Table 1:** Baseline characteristics (*n* = 231).

Characteristics	Value
Sex, *n* (%)	
Male	176 (76.19)
Female	55 (23.81)
Age (years)	
Range	3–18
Median (IQR)	12 (10–14)
Time pectus excavatum detected, *n* (%)	
At birth	14 (6.06)
At Childhood	152 (65.80)
At Puberty	65 (28.14)
Symptomatic, *n* (%)	
Chest tightness	25 (10.82)
Palpitation	6 (2.60)
Shortness of breath after exercising	17 (7.36)
No symptoms	183 (79.22)
Scoliosis, *n* (%)	62 (26.84)
Mild	58 (25.11)
Moderate	4 (1.73)
No. of bars, *n* (%)	
Modified Nuss procedure	199
One bar	187 (93.97)
Two bar	12 (6.03)
Traditional Nuss procedure	32
One bar	30 (93.75)
Two bar	2 (6.25)
Length of postoperative hospitalization (days)	
Range	3–18
Median (IQR)	6 (5–8)
Complications, *n* (%)	20 (8.65)
Pneumothorax	7 (3.03)
Hydrothorax	1 (0.43)
Atelectasis	1 (0.43)
Wound infection	11 (4.76)

### 2.2 Classifications and definitions

Patients were divided into a modified Nuss procedure group (MP group) and a traditional Nuss procedure group (TP group) according to the surgical approach. Based on the number of bars implanted, patients were divided into two groups: the Single Bar Group (SB group) and the Double Bar Group (DB group).

Pectus excavatum was defined as an anatomical depression of the chest wall, diagnosed based on medical history and physical examination of anterior chest wall deformity (16). The severity of the deformity was assessed using the Haller index (HI), which was calculated from CT scans and/or chest radiographs (17). Indications for surgical treatment included the following criteria (18):

- HI > 3.25;
- Shortness of breath or exercise intolerance;
- Abnormal pulmonary function tests;
- Mitral valve prolapse, bundle branch block, or other cardiac abnormalities related to pectus excavatum;
- Significant psychosocial distress;
- Cardiac or pulmonary compression evidenced by CT or echocardiography;
- Progression of the deformity;
- Previous failed repair.

The first five items were considered major criteria, while the latter three were minor criteria (19). In this study, surgical candidates were required to meet at least two of the above criteria, including at least one major criterion.

### 2.3 Surgical techniques

**Figure 3:**
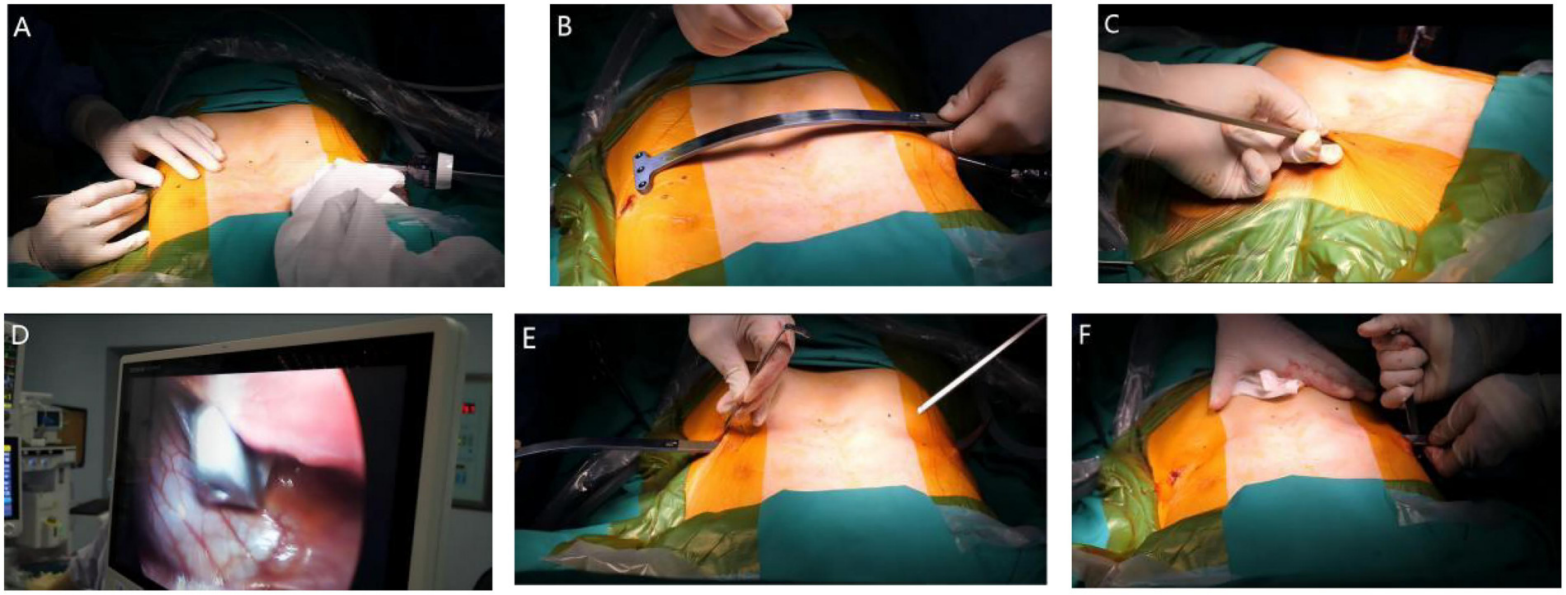
Key intraoperative steps. **(A)** shows the skin incision made at the premarked site. **(B–D)** show that under thoracoscopic guidance, the bar introducer was inserted through the intercostal muscles into the left thoracic cavity, advanced through the anterior mediastinal tissue and pericardium until it reached the right thoracic cavity, and was carefully guided until its tip was visualized at the right-side exit point. **(E,F)** show the introducer was completely withdrawn, the bar was left in position, and then secured on the right side with a stabilizer.

Routine anesthesia and disinfection were performed. Three marking points were made to indicate the entry point of the bar, the lowest point of the chest depression, and the exit point of the bar. The size of the bar was selected in advance based on measurements of chest width. Approximately 2 cm long incisions were made bilaterally near the mid-axillary line. The imaginary line connecting the midpoints of the two incisions passed through the lowest point of the pectus excavatum. Subsequently, the subcutaneous tissue was dissected using mosquito forceps to create a subcutaneous tunnel down to the fascia of the pectoralis major muscle. Stainless steel wires (ETHICON, LLC) were then passed around the superior and inferior ribs on both sides, with the intercostal entry point for the bar located between them. If pre-placement of the wire is considered to hinder the procedure, the wire can alternatively be sutured after insertion of the bar. A trocar was inserted adjacent to the entry point on the right side through the incision, and carbon dioxide was insufflated into the thoracic cavity. Under thoracoscopic guidance, the bar introducer was inserted through the intercostal muscles into the left thoracic cavity, advanced through the anterior mediastinal tissue and pericardium until it reached the right thoracic cavity, and was carefully guided until its tip was visualized at the right-side exit point. No flipping of the bar was required. A sternal elevator was not routinely utilized during standard primary procedures. However, in selected complex cases—specifically redo surgeries or patients with a history of prior cardiac surgery—where dense retrosternal adhesions to the pericardium were present, subxiphoid dissection was meticulously performed to release the adhesions, followed by sternal suspension to safely elevate the sternum before instrument insertion. Once the introducer was completely withdrawn, the bar was left in position and secured on the right side with a stabilizer. The bar was then fixed bilaterally to the ribs using the pre-placed steel wires. Routinely, bilateral fixation was achieved using two steel wire on each side: on one side, the wires are passed through the holes of the bar and secured to the adjacent upper and lower ribs; on the contralateral side, the wires are passed through the holes of the stabilizer and are similarly secured to the upper and lower ribs. For the double-bar technique, each bar was secured bilaterally with one steel wire. To further stabilize the construct, a bridging wire was passed through the adjacent holes of the superior and inferior bars on both sides to link them together, providing enhanced resistance against bar displacement. A small thoracic drainage tube was inserted through the trocar to evacuate the carbon dioxide from the pleural space. Finally, the subcutaneous tissue and skin were closed with sutures. Figure 3 illustrates several key intraoperative steps.

### 2.4 Postoperative management

All patients were monitored in the Cardiac Intensive Care Unit (CICU) for at least one day postoperatively. Prophylactic antibiotics and analgesics were administered to all patients. Patients were instructed to maintain a supine position and avoid lateral decubitus positions to prevent pressure on the chest. Early ambulation was encouraged to reduce the risk of complications such as pressure sores and pulmonary infections. Chest radiography was performed on the day of surgery to verify the position of the bar and evaluate the surgical outcome. A structured rehabilitation program was implemented for three consecutive days postoperatively to facilitate early recovery, including joint mobilization exercises, sensory integration therapy, and therapeutic exercises. To prevent any confounding effects on the evaluation of postoperative pain, patients in both the MP and TP groups received an identical, standardized pain management protocol and rehabilitation pathway.

### 2.5 Statistical analysis

Categorical variables are presented as frequency (n) and percentage (%). The Shapiro–Wilk test was used to assess the normality of the data. Continuous variables that followed a normal distribution are expressed as mean ± standard deviation, and comparisons between groups were performed using the t-test. Continuous variables that did not follow a normal distribution are expressed as median (interquartile range, IQR), and group comparisons were conducted using nonparametric tests. A *p*-value of less than 0.05 was defined as statistically significant. All statistical analyses were performed using SPSS Statistics 24 (SPSS Inc.).

### 2.6 Follow-up

All patients were instructed to attend regular postoperative follow-up visits at the outpatient clinic at 1, 3, and 6 months, as well as at 1, 2 and 3 years after surgery. At our center, the timing of bar removal is determined based on the individual patient's condition and our clinical experience. During each visit, a thorough physical examination was performed by the surgeon, and chest and spinal radiography were routinely conducted to evaluate the position of the bar and the spinal morphology.

## 3 Results

### 3.1 Preoperative characteristics

Over a period of 2.5 years, a total of 231 patients with pectus excavatum underwent surgery. Forty-eight patients had clinical symptoms, including chest tightness, palpitations, and shortness of breath after exercising. Echocardiography and CT revealed varying degrees of cardiac compression, cardiomegaly, or impaired ventricular diastolic function in 132 patients. Abnormal electrocardiogram findings were observed in 71 patients, including complete or incomplete right bundle branch block, T-wave changes, premature ventricular contractions, ventricular preexcitation, atrioventricular block, and right axis deviation. Scoliosis was identified in 62 patients, with 58 cases classified as mild and 4 as moderate.

### 3.2 Operative and postoperative characteristics

**Figure 4:**
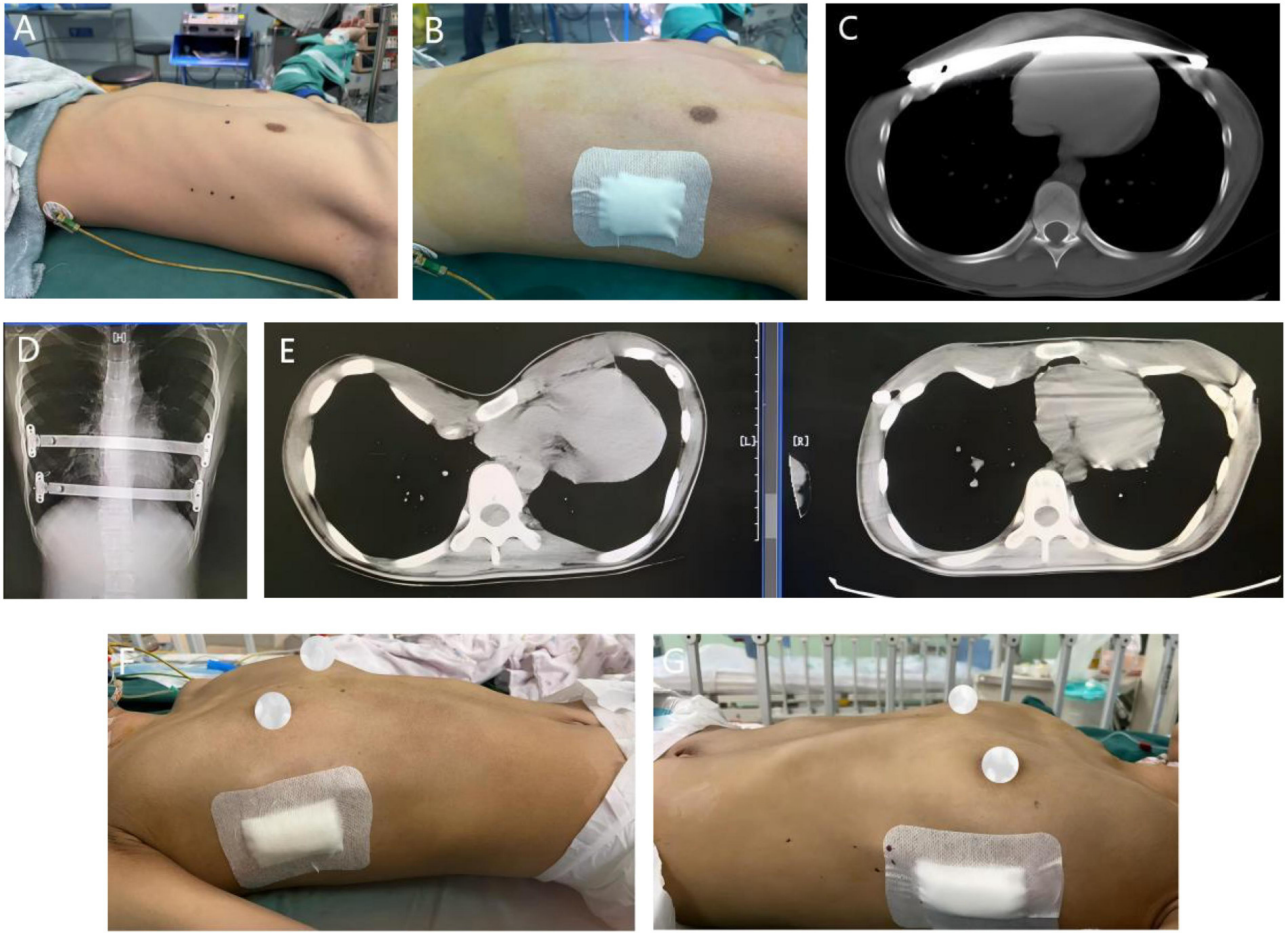
**(A,B)** show preoperative and postoperative chest photographs of a male patient. **(C)** shows postoperative chest CT imaging. **(D,E)** show intraoperative placement of double bars in a female patient with severe pectus excavatum, along with preoperative and postoperative radiographic images. **(F,G)** show postoperative chest photographs.

No intraoperative life-threatening events such as cardiopulmonary injury, cardiac arrest, or life-threatening hemorrhage occurred among the 231 patients. A total of 231 matched patients were included in the subsequent analysis. Table 2 shows that significant differences were found between the MP and TP groups in operation time [median 65.00 min (IQR 55.00–65.00 min) vs. 87.50 min (80.00–93.75 min), *P* < 0.001], postoperative pain score [6.00 (5.00–6.00) vs. 6.00 (5.00–7.00), *P* < 0.05] and postoperative hospital stay [6.00 days (5.00–7.00 days) vs. 9.0 days (7.25–11.00 days), *P* < 0.001]. In addition, Table 3 shows that significant differences were observed between the SB and DB groups in age [12.00 years (10.00–13.00 years) vs. 4.10 years (3.56–4.90 years), *P* < 0.001], operation time [65.00 min (55.00–65.00 min) vs. 95.00 min (90.00–101.25 min), *P* < 0.001] and postoperative hospitalization time [6.00 days (5.00–7.00 days) vs. 7.50 days (6.00–11.00 days), *P* < 0.05]. Each case had an improvement in Haller index. Figure 4 show the comparative thoracic contour before and after the thoracoscopy-assisted modified Nuss procedure in patients with pectus excavatum.

**Table 2:** Comparison of preoperative and postoperativ outcomes between MP group and TP group.

Variable	MP group (*n* = 199)	TP group (*n* = 32)	*P* values
Age (years), meidan (IQR)	12.00 (10.00–14.00)	12.00 (10.00–14.00)	0.670
Preoperative Haller Index, meidan (IQR)	3.65 (3.35–4.33)	3.60 (3.37–3.98）	0.671
Postoperative Haller Index, meidan (IQR)	2.67 (2.44–2.93)	2.72 (2.72–2.92)	0.556
Operation time (min), meidan (IQR)	65.00 (55.00–65.00)	87.50 (80.00–93.75)	<0.001
Postoperative pain score, meidan (IQR)	6.00 (5.00–6.00)	6.00 (5.00–7.00)	0.011
Postoperative hospitalization time (day), meidan (IQR)	6.00 (5.00–7.00)	9.0 (7.25–11.00)	<0.001
Complications, *n* (%)	17 (8.54)	3 (9.38)	>0.05
Pneumothorax	5	0	
Hydrothorax	0	1	
Atelectasis	1	0	
Wound infection	10	1	

**Table 3:** Comparison of preoperative and postoperative outcomes between SB group and DB group.

Variable	SB group (*n* = 217)	DB group (*n* = 14)	*P* values
Age (years), meidan (IQR)	12.00 (10.00–13.00)	14.5 (13.75–15.00)	<0.001
Preoperative Haller Index, meidan (IQR)	3.63 (3.35–4.25)	3.98 (3.48–4.58）	0.117
Postoperative Haller Index, meidan (IQR	2.67 (2.44–2.90)	2.84 (2.62–3.13)	0.055
Operation time (min), meidan (IQR)	65.00 (55.00–65.00)	95.00 (90.00–101.25)	<0.001
Postoperative pain score, meidan (IQR)	6.00 (5.00–6.00)	6.00 (5.75–7.00)	0.257
Postoperative hospitalization time (day), meidan (IQR)	6.00 (5.00–7.00)	7.50 (6.00–11.00)	0.003
Complications, *n* (%)	18 (8.29%)	2 (14.29)	>0.05
Pneumothorax	7	0	
Hydrothorax	0	1	
Atelectasis	1	0	
Wound infection	10	1	

### 3.3 Follow-up and clinical outcomes

The follow-up period ranged from 6 to 30 months. During follow-up, seven pediatric cases developed pneumothorax in the early postoperative phase and were successfully managed conservatively. One child presented with bilateral pleural effusion shortly after surgery and improved after closed thoracic drainage. Another case was complicated by right-sided atelectasis during the early recovery period and recovered with conservative management. Eleven patients developed surgical site infections, among whom four required reoperation due to prolonged wound healing issues. All patients achieved satisfactory chest wall appearance postoperatively. Among the 62 patients with concomitant scoliosis, longitudinal follow-up revealed that the spinal curvature remained stable in 51 cases (82.3%). In the 8 patients (12.9%) who experienced curvature progression, intervention was tailored to severity: three patients with moderate scoliosis were treated with orthopedic braces and showed subsequent improvement, while five patients with mild scoliosis remained under close observation without specific intervention. Spontaneous improvement of the spinal curve was noted in 3 patients (4.8%). No specific conservative management was initiated during the acute hospitalization phase for these patients.

## 4 Discussion

The reported prevalence of pectus excavatum currently ranges from 1 in 700 to 1 in 1,000 live births, with a higher incidence in males than females (1). Preoperative auxiliary examinations primarily include chest CT, electrocardiography, echocardiography, and pulmonary function tests. In recent years, cardiopulmonary exercise testing (CPET) has been increasingly utilized. Jaroszewski et al. conducted CPET on 392 pectus excavatum patients both preoperatively and postoperatively, demonstrating significant improvements in several parameters following minimally invasive Nuss surgical repair, including peak VO₂, oxygen consumption, and maximum ventilation volume (20). Various surgical techniques exist for correcting this chest wall deformity, with the Ravitch and Nuss procedures being the two most widely applied methods today. The Ravitch procedure was first introduced in 1949, while the Nuss procedure was adopted in 1987 (21). However, as previously mentioned, these surgical techniques are also associated with several limitations.

The thoracoscopy-assisted modified Nuss procedure introduced by Dr. Guoqing Li eliminates the need for bar flipping, thereby reducing intraoperative trauma and improving safety. Eliminating the bar flipping step not only streamlines the surgical workflow but, more crucially, averts transient compression of the heart and pericardium during rotation, thereby mitigating the risk of circulatory instability. Furthermore, this modification reduces mechanical shear forces on the intercostal muscles and parietal pleura, effectively safeguarding the intercostal neurovascular bundles. This reduction in local tissue trauma may subsequently contribute to a further decrease in postoperative pain levels for the patients.The MP group had lower postoperative pain scores and a shorter postoperative length of hospital stay than the TP group, with statistically significant differences. The thoracoscopy-assisted modified Nuss procedure also eliminates the need for intraoperative bending and facilitates easier assembly, contributing to reduced operative time. The operative time was also shorter in the MP group than in the TP group, and the difference was statistically significant. Furthermore, our center has observed a low rate of postoperative complications, most of which are mild in nature. The postoperative complication rate in our study group was 8.65%, which is relatively lower compared to the 12.28% rate reported by Kelly for the conventional Nuss procedure (22).

**Figure 5:**
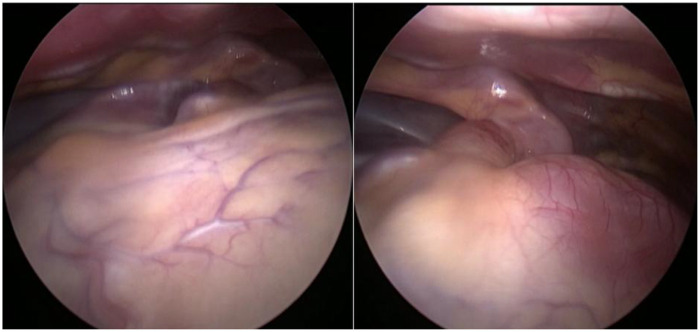
The intraoperative thoracoscopy revealed an absence of the left pericardium.

Some studies indicate that the repeated passage of the introducer and bar may pose risks of pulmonary or cardiac injury. Therefore, many thoracic surgeons emphasize the necessity of thoracoscopic assistance to ensure safety and minimize complications (23, 24). In one case in our series, intraoperative thoracoscopy revealed the absence of the left pericardium (Figure 5). Without thoracoscopic guidance, the risk of cardiac injury in this patient would have been significantly higher. Importantly, the use of thoracoscopy does not prolong operative time nor require additional incisions, and its routine adoption is strongly recommended.

Bar fixation is a critical factor in ensuring surgical outcomes. The novel bar design incorporates screw holes that allow for stable fixation using screws and locking clips. Furthermore, both ends of the bar are additionally stabilized with wires to enhance overall stability. No instances of bar displacement occurred in this study, which reduced the reoperation rate. We observed that larger bars may contribute to a higher incidence of incision-related complications, such as surgical site infection, potentially due to excessive pressure on the skin and subcutaneous tissue. Cutaneous redness and swelling, which may be related to the local rejection of plates, can be effectively alleviated with corticosteroid treatment when present.

When a single bar does not achieve the desired aesthetic correction, an additional bar is implanted through the same bilateral incisions. In our study, two bars were implanted in twelve patients with pectus excavatum, and no patient required a third bar. Experience has shown that patients requiring dual-bar correction are often tall and slender with a flattened chest wall. Although a statistically significant age difference was observed between the dual-bar and single-bar groups, there was no significant difference in preoperative HI, indicating that the HI is not the decisive factor for implanting two bars. The decision is primarily based on the intraoperative assessment by the surgeon and the aesthetic expectations of the patient and family. The second bar is typically placed in the second or third intercostal space and is smaller in size compared to the primary bar. We employ a figure-of-eight wiring technique to fix the bars and additionally suture them to the ribs and chest wall muscles to reduce the risk of bar displacement. Due to the larger incision and increased tissue exudate in dual-bar cases, the placement of a subcutaneous negative-pressure drainage device is recommended. The operation time and length of postoperative hospital stay were significantly longer in the DB group than in the SB group. Empirical evidence suggests that patients with dual bars experience prolonged postoperative pain, requiring an extended recovery period. The overall incidence of postoperative complications in this study group was low, and the statistical results showed that there was no significant difference in the incidence of postoperative complications between the SB group and the DB group.

Postoperative pain management is a priority during the recovery period. At our institution, intraoperative intercostal nerve blockade with ropivacaine is performed, followed by postoperative analgesia with a sufentanil plus dolasetron patient-controlled analgesia pump for 48 h, supplemented by oral ibuprofen and rectal diclofenac potassium for pain relief. Specifically, during the first three postoperative days, patients routinely receive oral ibuprofen twice daily, combined with rectal diclofenac potassium administered at bedtime. After this initial three-day period, analgesics are transitioned to an as-needed basis depending on the patient's pain assessment. For patients experiencing more significant breakthrough pain, oral acetaminophen tablets are added to the regimen. The overall pain control in this study was acceptable. Numerous techniques have been developed to improve postoperative analgesia, including epidural analgesia, multimodal analgesia, and programmable elastomeric pumps. Several researchers have reported that intercostal nerve cryoablation (INC) is an effective option for pain management (25). Rettig et al. also demonstrated that intercostal nerve cryoablation is a novel approach that can reduce hospital length of stay, improve pain control, and decrease overall costs. They recommended incorporating INC into the standard postoperative pain management protocol for patients with pectus excavatum (26). Second, early postoperative rehabilitation is equally crucial. At our center, an individualized rehabilitation program is initiated on the first day after surgery. This includes joint mobilization training, sensory integration therapy, and kinesiotherapy. Joint mobilization exercises help establish a foundational range of motion for subsequent kinesiotherapy. Sensory integration therapy enhances neuromuscular control and improves coordination during motor training. Kinesiotherapy serves to consolidate the long-term effects of both joint and sensory interventions. Besides, starting on the first postoperative day, patients are not only guided through active limb movements and early out-of-bed mobilization, but they are also placed on a strict respiratory training regimen. This pulmonary rehabilitation includes guided deep breathing exercises, instruction on effective coughing techniques for airway clearance, and the routine use of incentive spirometry (or balloon-blowing exercises for younger children) to promote rapid lung re-expansion and prevent pulmonary complications such as atelectasis.

This study has several limitations. First, as a retrospective single-center study, it was challenging to control for all potential variables to minimize selection bias. Second, the follow-up period was limited, and the sample size was relatively small. This study only assessed the short- and mid-term outcomes of the thoracoscopy-assisted modified Nuss procedure; further investigation is required to evaluate long-term efficacy after bar removal. Third, the modified technique demands a high level of proficiency and extensive experience from the surgeon. Future large-scale, multicenter, prospective studies are needed to validate the safety and efficacy of the modified thoracoscopy-assisted Nuss procedure.

## 5 Conclusions

This single-center experimental study demonstrates that, despite certain limitations, compared to traditional Nuss procedure, the thoracoscopy-assisted modified Nuss procedure achieved a comparable correction of the HI while offering shorter operation time, lower postoperative pain score, and shorter postoperative hospital stay, which may be suitable for pediatric patients with pectus excavatum. Further evaluation will be discussed in future studies.

## Data Availability

The raw data supporting the conclusions of this article will be made available by the authors, without undue reservation.
